# A State of Unrest. A New Movement on Foot

**Published:** 1917-09-22

**Authors:** 


### NURSING AFFAIRS IN IRELAND.

### A State of Unrest. A New Movement on Foot.

PURSUING the subject of the Economics of Nursing, I learn that a new movement is on foot, independent both of the College of Nursing and of the Irish Nursing Board. The object in view is to establish an Irish Nurses' League. The initiator of the scheme is Miss Kearns, a trained nurse, and secretary of the Irish Nurses' Insurance Society, formed to meet the requirements of the National Health Insurance Act. Miss Kearns is a lady of ability and of independent mind, a past if not a present member of the Irish Nurses' Association, and in addition, strange as it may or may not appear, a member of the committee of the Irish Nursing Board. I understand also that a part of the League's programme is to establish an Irish nurses' newspaper. At present the scheme is more or less *in nubibus*, and my object in making reference to it now is to show that in the nursing world here there is at the moment considerable unrest—pent-up discontent seeking an outlet—combined with a lack of faith in any existing institution. This is a fact which had better be faced, as nothing can ever be gained by ignoring the issue.

### THE REVOLT OF THE RANK AND FILE.

There is no room for doubt that the policy of the Irish Nurses' Association, consistently pursued for many years, of ignoring the rank and file of the profession, has caused revolt, nor is there any doubt that the root cause of the trouble is economic. There must be better pay and better conditions of service, and, sooner or later, Irish hospital boards will be forced to realise that the employment of nurses' earnings and the sweating of probationers is not a policy that can continue, despite the fact that the objective is charity. Almost every hospital in Dublin gets its nursing done at present in return for the boarding and lodging of the nursing staff, or thereabouts. The fees of probationers and the revenue derived from private nursing fees about balance the salary charge, the trained staff receiving in a majority of cases what can only be described as a pittance. Nurses would be lacking in intelligence if they failed to appreciate that in every other domain of women's work the scale of wages paid in comparison with theirs is lavish.

### A FLATTERING TESTIMONIAL TO THE COLLEGE.

If I may say so, I think it is premature at this juncture to start a new faction. The College of Nursing, so far as Ireland is concerned, has scarcely had time to find its feet, and by all the laws of Nature a division of forces is bound to have a weakening effect. If the founders and propagandists of the League are wise, they will continue to maintain their independence at least until they see how the College develops. It has begun its life with a programme which has dazzled the Irish Nursing Board, its avowed enemy, and this, I venture to suggest, is its most flattering testimonial. Of course the starting of an Irish nurses' journal sounds well, but I may take the opportunity of reminding the promulgators of the desire of Job that his adversary would write a book! I could wish my adversary, whomsoever he may be, no worse fate just now than that he should start a newspaper. By the time that he has paid his printers, and broken his heroic heart in trying to find advertisers, he will have little fighting spirit left. It is not a business proposition.

### A BUSINESS PROPOSITION WANTED.

Nevertheless we want a business proposition in Ireland. We want a forward propaganda which will consist, fundamentally and throughout, of a bread-and-butter argument. The Irish nurse has never had a champion, and the people who had the duty of organising and helping the profession during the present generation have a barren record. Of this there is no more convincing proof than the fact that in their narrowness and ignorance they effectually prevented the Midwives Act from extending to this country. They were persuaded that in some subtle way they were going to be "done." The glory of the Rotunda Hospital they regarded as calculated to eclipse all other institutions, and so, in an evil hour, they held aloof. Hence I regard Miss Kearns' lack of faith as perfectly natural.

### A POLICY OF ISOLATION IS NOT PATRIOTISM.

An attempt is being made in some quarters to link up patriotism with the new movement, and a cry of this kind goes a long way in Ireland. It is an illusion. True patriotism and isolation are not concurrent forces. Nations do not progress by confining their energies within their own borders, but rather by extending their commerce and by establishing an *entente cordiale* with the outer world. Moreover, the nurse who works always and plays little is mainly concerned with providing butter for her bread, and with getting also a little jam if possible. In any case, with all due reverence for patriotism, real or falsely so called, it adds little to the annual income of working men and women. No merchant curtails his business from patriotic motives. On the contrary, his constant endeavour is to extend his sphere of influence. It is to be hoped, therefore, that the rank and file of Irish nurses will not be lured into a *cul-de-sac*, at all events until they have closely investigated all the circumstances, and weighed carefully in the balance by what machinery they can best further their material interests. That they are alive and alert is a matter for congratulation, and that there is an underlying fear of being "doped" is not a matter for wonderment.

But no man or woman of intelligence will contend that a divided house makes for progress. Look at Russia, a spectacle calculated to make angels weep!

IERNE.

